# *In silico* secretome analysis approach for next generation sequencing transcriptomic data

**DOI:** 10.1186/1471-2164-12-S3-S14

**Published:** 2011-11-30

**Authors:** Gagan Garg, Shoba Ranganathan

**Affiliations:** 1 Dept. of Chemistry and Biomolecular Sciences, Macquarie University, Sydney NSW 2109, Australia; 2 Dept. of Biochemistry, Yong Loo Lin School of Medicine, National University of Singapore, 8 Medical Drive, Singapore 117597

## Abstract

**Background:**

Excretory/secretory proteins (ESPs) play a major role in parasitic infection as they are present at the host-parasite interface and regulate host immune system. In case of parasitic helminths, transcriptomics has been used extensively to understand the molecular basis of parasitism and for developing novel therapeutic strategies against parasitic infections. However, none of transcriptomic studies have extensively covered ES protein prediction for identifying novel therapeutic targets, especially as parasites adopt non-classical secretion pathways.

**Results:**

We developed a semi-automated computational approach for prediction and annotation of ES proteins using transcriptomic data from next generation sequencing platforms. For the prediction of non-classically secreted proteins, we have used an improved computational strategy, together with homology matching to a dataset of experimentally determined parasitic helminth ES proteins. We applied this protocol to analyse 454 short reads of parasitic nematode, *Strongyloides ratti*. From 296231 reads, we derived 28901 contigs, which were translated into 20877 proteins. Based on our improved ES protein prediction pipeline, we identified 2572 ES proteins, of which 407 (1.9%) proteins have classical N-terminal signal peptides, 923 (4.4%) were computationally identified as non-classically secreted while 1516 (7.26%) were identified by homology to experimentally identified parasitic helminth ES proteins. Out of 2572 ES proteins, 2310 (89.8%) ES proteins had homologues in the free-living nematode *Caenorhabditis elegans* and 2220 (86.3%) in parasitic nematodes. We could functionally annotate 1591 (61.8%) ES proteins with protein families and domains and establish pathway associations for 691 (26.8%) proteins. In addition, we have identified 19 representative ES proteins, which have no homologues in the host organism but homologous to lethal RNAi phenotypes in *C. elegans*, as potential therapeutic targets.

**Conclusion:**

We report a comprehensive approach using freely available computational tools for the secretome analysis of NGS data. This approach has been applied to *S. ratti* 454 transcriptomic data for *in silico* excretory/secretory proteins prediction and analysis, providing a foundation for developing new therapeutic solutions for parasitic infections.

## Background

The secretome of an organism is defined as the subset of proteins secreted by the cell [1]. This subset of proteins is usually known as excretory/secretory (ES) proteins [2], plays an important role in producing clinical infections in the host organism. ES proteins are the choice of new therapeutic solutions for different clinical infections, especially in the case of parasitic infections [3,4] because these proteins are present at the host-parasite interface and act as immunoregulators to host immune recognition for parasite survival inside the host organism [5].

Transcriptomic data is the representation of actively expressed genes in a cell at any given time. Earlier transcriptomic studies were based on generation of expressed sequence tags (ESTs) generated at different stages of an organism using traditional Sanger sequencing. These studies were restricted to the analysis of a few thousand ESTs at a time. Recent technological improvements in cDNA sequencing, using next generation sequencing (NGS) platforms, are able to generate millions of reads, to record the transcript profile of an organism at a given developmental stage. The read length generated through NGS is quiet short (50-400 bases) as compared to traditional Sanger sequencing (800-1000 bases). Thus, the assembly of shorter reads is challenging in terms of computational power and resources needed. These reads are assembled into long consensus sequences (clusters) known as contigs using assemblers such as ABySS [6], Velvet [7] and MIRA [8], which have been reviewed in a recent study [9]. ABySS and Velvet provide good results for genome assembly, while MIRA is very well tested for handling *de novo* transcriptome assembly [10]. Since the genomes of only a very few parasitic nematodes are currently available, *de novo* assemblers such as MIRA are the only option for NGS data from these neglected organisms.

Recently, NGS platforms have been used to generate large amounts of transcriptomic data for different organisms, including several helminth parasites like *Fasciola gigantica *[11], *Fasciola hepatica*[12], *Trichostrongylus colubriformis*[13], *Oesophagostomum dentatum*[14], *Haemonchus contortus *[15], *Dictyocaulus viviparus*[16], *Necator americanus *[17], *Clonorchis sinensis*[18], *Opisthorchis viverrini *[18] and *Teladorsagia circumcinta*[*19*]. Here, NGS data has been assembled with CAP3 alone [14,16] or with MIRA followed by CAP3 [12,18], based on combinations of assemblers performing better in a recent study [10]. However, none of these studies have extensively covered ES protein prediction and further analysis, for identifying therapeutic targets.

ES proteins were once considered to be secreted only through conventional secretion pathways, using N-terminal signal peptide signatures, but there are now many proteins which are found to be secreted by non-classical secretory pathways [20]. Usually non-classical secretory proteins are predicted through SecretomeP [21], which is the most widely used tool for non-classical secretory proteins. However in case of parasites, SecretomeP is not able to completely predict non-classical secretory proteins, as shown in the study of *Brugia malayi*[*22*]. Hence, a novel approach to identifying non-classically secreted proteins is required for comprehensive secretome analysis.

Transcriptomic data has been used extensively for the prediction of ES proteins in parasitic helminth studies [23]. EST2Secretome, a computational prediction and annotation pipeline for ES proteins from our group, was designed to handle ESTs from Sanger sequencing and currently has the following limitations: (i) assembly of short reads, (ii) prediction of non-classical secretory proteins and (iii) pathway mapping using KOBAS [24,25], which contains pathways that are not regularly updated.

In the present study, we have developed an updated computational approach for the prediction and annotation of ES proteins using NGS transcriptomic data overcoming the limitations of the earlier EST2Secretome pipeline. We have developed a robust assembly protocol for NGS data. In order to identify non-classically secreted proteins that are missed by SecretomeP, we have also compiled a dataset of experimentally determined ES proteins of parasitic helminths for homology-based prediction (details in the Methods section). Additionally, we have replaced KOBAS with KAAS [26], for efficient and up-to-date pathway identification.

We applied our approach to ~0.3M 454 transcriptomic reads for a parasitic nematode, *Strongyloides ratti*, which is a gastro intestinal nematode that infects rats, comprehensively reviewed by Viney [27] and is a Clade IV parasite [28]. Genome data is available only for the free living nematodes, *C. elegans*[*29*] and *C. briggsae*[*30*] from Clade V, which is adjacent to Clade IV and for a parasite, *Brugia malayi*[*31*] from Clade III, which is not similar to Clade IV parasites, whereas limited transcriptomic and proteomic data from experimental studies are available for several helminth parasites. As such, a BLASTX against a reference organism, as proposed recently [32] will not provide comprehensive annotation results, unless the fully annotated proteome of a very similar organism is available.

In adult phase, *S. ratti* is present in both parasitic (females only) and free living forms (male and female) [27]. Eggs produced by parasitic females develop into free living males, free living females and parasitic females by different larval stages. Our dataset is derived from the adult nematode, which includes parasitic and free living forms (sequencing details in the Methods section). The NGS data has been clustered and translated into proteins and ES proteins predicted using a series of computational tools, augmented by homology matching to our in-house dataset of experimentally determined parasitic helminth ES proteins. Predicted ES proteins have been annotated functionally in terms of protein families, domains and biochemical pathways. ES proteins have also been compared with proteomic data of the host (rat) and other nematodes, with an emphasis on the best characterized nematode, *C. elegans*. Such annotation techniques have enabled us to identify 19 novel targets, matching to lethal RNAi phenotypes in *C. elegans*, which could be considered in the development of future therapeutic strategies.

## Methods

### cDNA sequencing data sets

For this study, *S. ratti* cDNA sequencing data from the University of Liverpool [33] is used. cDNA libraries were prepared from adult helminths, comprising a mixture of parasitic females, free-living males and free-living females. Sequencing was performed using 454-FLX platform (Roche diagnostics). The pyrosequencing procedure used to prepare this dataset is described elsewhere [34].

### Components of computational approach

**Figure 1:**
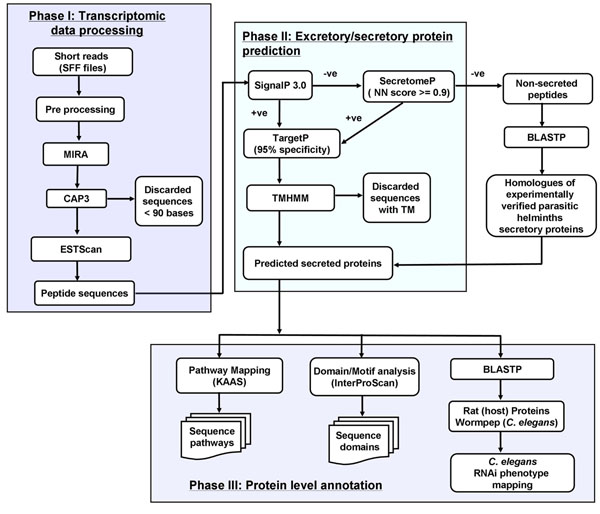
**Bioinformatics workflow for secretome analysis.** Bioinformatics workflow comprising Phase I (pre-processing and assembly), II (prediction of excretory/secretory proteins) and III (Protein-level annotation) were augmented by homologue identification from nematodes as well as parasitic nematodes, using specialized databases.

Our approach to predict and annotate ES proteins is divided into three phases, shown in Figure 1, corresponding approximately to those in EST2Secretome [23]. EST2Secretome was developed with the aim to predict and annotate ES proteins from ESTs (generated mainly using Sanger sequencing) mainly from parasitic nematodes. Now with the use of NGS, the input sequence data has changed considerably in terms of read length and number; necessitating modifications to tackle NGS data as well reliably predict non-classical protein secretion and use updated annotation tools.

#### Phase I: extraction and assembly of data

FASTA and associated quality files were extracted from SFF file along with clipping of sequence adapters using the sff_extract software [35]. Extracted data from sff files is first assembled using the MIRA [8] (V3.2.0rc1) assembler using quality information. MIRA is our preferred assembler as it is an open source tool which is considered reliable for data from different NGS platforms [8] and it has been very well tested in other parasitic helminth transcriptomic studies [12,18]. For this dataset, we have used MIRA, ABYSS and Velvet, compared with Newbler (data not shown), MIRA giving the longest contigs. Contigs generated by MIRA are further passed to the Contig Assembly Program (CAP3) [36], to extend the MIRA assembly. This is in accord with an earlier study which suggests that serial assembly from two assemblers can improve the quality of the assembly [10]. Second order contigs generated using CAP3 are combined with MIRA contigs, to be conceptually translated into putative proteins using ESTScan [37].

#### Phase II: prediction of excretory secretory proteins

ES proteins were predicted using a combination of four tools, SecretomeP [21], SignalP [38], TargetP [39] and TMHMM [40]. SignalP is used for the prediction of classical secretory proteins, while SecretomeP predicts non-classical secretory proteins. TargetP is for the prediction of mitochondrial proteins and TMHMM identifies transmembrane proteins. Firstly, the proteins generated from ESTScan are passed to SignalP for prediction of classical secreted proteins. All the proteins, which are predicted as non-secretory (proteins having D score and signal peptide probability less than 0.5) are then passed to SecretomeP for prediction of non-classical secretory proteins. Proteins which obtain neural network (NN) score of greater than or equal to 0.9 are considered as non-classical secretory proteins. All the classical and non-classical secretory proteins are merged together and then scanned by TargetP. Proteins predicted as mitochondrial proteins by TargetP are omitted out from the set of predicted ES proteins and passed to TMHMM. Finally the proteins which are predicted to have no transmembrane helices are considered as ES proteins.

In addition to standard computational approaches for the prediction of ES proteins, we compiled a list of 1080 ES protein sequences of parasitic helminths (*Brugia malayi*, *Teladorsagia circumcinta*, *Schistosoma mansoni*, *Ancylostoma caninum*, *Schistosoma japonicum*, *Clonorchis sinesis* and *Fasciola hepatica*) from the literature [22,41-49]. A homology-based search with BLASTP [50] is used to further extract ES proteins from proteins which are predicted to be non-secretory by SecretomeP.

The results from computational tools are combined with those from BLAST searches, for functional annotation and analysis in Phase III.

#### Phase III: annotation and comparative analysis of ES proteins

All the predicted ES proteins are annotated using a number of tools. We used Interproscan [51] for protein domain and family classification. KAAS [26] is used for mapping ES proteins to KEGG pathways and to KEGG BRITE objects [52-54]. ES proteins are searched for sequence similarity against the Wormpep database (WS224) [55] for proteins similar to *C. elegans*. ES proteins are also searched for sequence similarity against rat (host) proteins and parasitic nematodes using BLASTP algorithm, to identify parasite-specific proteins. Comparative analysis of similarity of ES proteins with rat, parasitic nematodes and *C. elegans* proteins are analyzed using Simitri [56]. Proteins not homologous to the host (rat) proteome are further screened for RNAi phenotypes in *C. elegans.*

### Hardware specifications

All the programs used in this study were installed on a 16 CPU Linux cluster (2.4 GHz, Intel(R)Xeon(R) E5530, 32 RAM) running on ubuntu server operating system. The computer intensive steps are sequence assembly (MIRA, CAP3) and protein functional annotation mapping (Interproscan). All other programs will run efficiently on current desktop systems.

## Results

A semi-automated computational approach, incorporating three key components, was constructed. The different components of the workflow system (Figure 1) are linked using Perl, Python and bash shell scripts. This approach was applied to *S. ratti* 454 transcriptomic dataset to show its efficacy and utility.

### Extraction and assembly of *S. ratti* data sets

Initially 296231 short reads (69488625 bases) were extracted from the sff file with 234±62 bases (average length ± standard deviation), and a GC content of 39.7%. The *de novo* assembly from MIRA results in 33222 contigs, which were passed to CAP3 to get a more robust assembly, with a minimum sequence overlap length of 40 bases and an identity threshold of 90%. Using CAP3, we are able to achieve a maximum contig length of 3620 bases as compared to maximum contig length of 2607 bases by Newbler [34]. The CAP3 assembly results in 3056 second order contigs and 25845 MIRA contigs (not assembled further by CAP3). The difference in results using MIRA+CAP3 and Newbler are shown in Table 1. We consider 25765 (99.6%) contigs with a minimum length of 90 bases, discarding sequences yielding peptides <30 amino acids, for further secretory protein prediction and analyses. A total of 3056 second order contigs and 25765 contigs were conceptually translated into 20877 proteins by ESTScan.

**Table 1:** Comparison of results from different NGS assemblers

Assembler	No. of second order contigs	No. of contigs	Largest contig	Average length	N50*	N90*	Number of bases
MIRA [8] + CAP3 [29]	3056	25845	3620	402.36	406	253	11628536
Newbler [26]		25127	2607	407.11	409	252	10229510

*N50 refers to the length of the shortest contig such that the sum of contigs of equal length or longer is at least 50% of the total assembly size. While N90 refers to the length of the shortest contig such that the sum of contigs of equal length or longer is at least 90% of the total assembly size.

### Prediction of ES proteins

ES protein prediction is carried out in Phase II of the pipeline (Figure 1). Firstly, 407 (1.9%) proteins were predicted as classical secreted proteins using SignalP. The remaining 20470 (98.05%) proteins, which were predicted as non secretory by SignalP were processed by SecretomeP for prediction of non-classical secretory proteins. A total of 923 (4.4%) proteins were predicted as non-classical secretory proteins using SecretomeP. The classical and non-classical secretory proteins (1330, 6.3%) from these two programs were analyzed by TargetP for mitochondrial proteins. Only 18 proteins were predicted as mitochondrial proteins using TargetP at 95% specificity. These 18 proteins were removed from the set of 1330 secreted proteins while 1312 secretory proteins were passed to TMHMM for the prediction of transmembrane proteins. 256 proteins, predicted as transmembrane proteins having one or more transmembrane helices, were removed from the secretory protein dataset. A total of 1056 (5.05%) proteins were finally predicted as ES proteins from the computational prediction pipeline.

Proteins that were considered non-secretory by SecretomeP were matched to our in-house dataset of 1080 non redundant experimentally determined parasitic helminth proteins, using the BLASTP similarity search. We found an additional 1516 (7.26%) proteins similar to known ES proteins by this homology search approach. Thus, for annotation and analyses in Phase III, we compiled a total of 2572 ES proteins, which is 12.3% of our putative proteins. This dataset is a more comprehensive collection of ES proteins of *S. ratti*, compared to those reported by other *S. ratti* secretome studies [57,58].

### Annotation of *S. ratti* ES proteins

ES proteins are annotated based on protein families and domains using Interproscan and mapped to biochemical pathways using KAAS. Out of 2572 ES proteins predicted, we were able to annotate 1591 (61.8%) proteins with protein domains and families. The most represented Interpro terms are shown in Table 2 (complete results available from Additional file 1). We established pathway associations to 691 (26.8%) ES proteins. Among the most represented pathways are metabolic pathways, which are important for parasite survival inside the host. Predicted ES proteins are associated with important biological molecules, like enzymes, peptidases and protein kinases. The most represented KEGG BRITE objects and KEGG pathways are shown in Table 3 (full annotation available from Additional file 2) and Table 4 (full annotation available from Additional file 3).

**Table 2:** Top 15 most represented protein domains found in ES proteins using Interproscan

InterPro description	InterPro code	Number of ES proteins (%)
Protein Kinase like domain	IPR011009	126 (4.90)
Protein kinase, catalytic domain	IPR000719	114 (4.43)
Serine/threonine-protein kinase like domain	IPR017442	99 (3.85)
Serine/threonine-protein kinase domain	IPR002290	64 (2.49)
Serine/threonine-protein kinase active site	IPR008271	52 (2.02)
WD40 repeat like domain	IPR011046	40 (1.55)
WD40 repeat subgroup	IPR019781	39 (1.52)
WD40/YVTN repeat like domain	IPR015943	39 (1.52)
WD40 repeat	IPR001680	39 (1.52)
WD40 repeat domain	IPR017986	38 (1.47)
Tyrosine-protein kinase catalytic domain	IPR020635	37 (1.44)
WD40 repeat 2	IPR019782	37 (1.44)
Helicase C	IPR001650	35 (1.36)
NAD(P)-binding domain	IPR016040	29 (1.13)
Immunoglobulin-like fold	IPR013783	28 (1.09)

**Table 3:** Top 15 most represented KEGG pathways found in ES proteins predicted by KAAS

Pathway name	Number of ES proteins represented (%)
Metabolic pathways	109 (4.24)
Protein processing in endoplasmic reticulum	57 (2.22)
Ubiquitin mediated proteolysis	44 (1.71)
Wnt signalling pathway	29 (1.13)
Glycolysis / Gluconeogenesis	28 (1.08)
Spliceosome	28 (1.08)
Glutathione metabolism	26 (1.01)
Circadian rhythm - mammal	22 (0.85)
TGF- beta signalling pathway	22 (0.85)
RNA transport	20 (0.77)
Endocytosis	20 (0.77)
Purine metabolism	19 (0.74)
Phagosome	19 (0.74)
Proteasome	18 (0.70)
Drug metabolism	17 (0.66)

**Table 4:** Top 15 most represented KEGG BRITE objects found in ES proteins predicted by KAAS

BRITE object	Number of ES proteins represented (%)
Enzymes	282 (10.96)
Spliceosome	49 (1.90)
Chaperons and folding catalysts	44 (1.71)
Peptidases	44 (1.71)
Protein kinases	43 (1.67)
Ubiquitin system	37 (1.44)
Chromosome	34 (1.32)
Cytoskeleton proteins	27 (1.05)
DNA repair and recombination proteins	21 (0.82)
GTP-binding proteins	19 (0.74)
Proteasome	18 (0.70)
Transcription factors	17 (0.66)
Ribosome biogenesis	16 (0.62)
Translation factors	11 (0.43)
DNA replication proteins	9 (0.35)

### Comparative analysis of *S. ratti* ES proteins with other organisms

**Figure 2:**
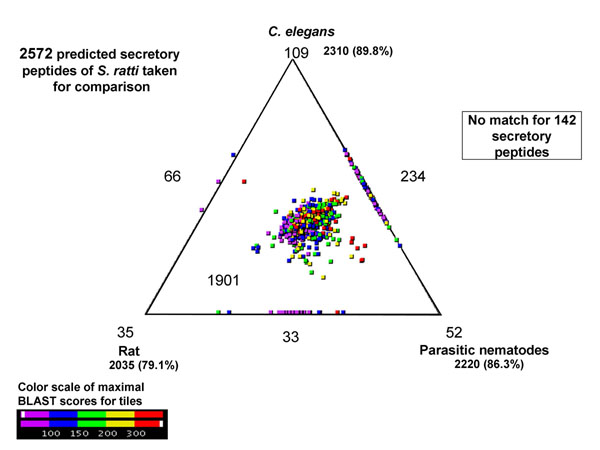
**Comparison of *S. ratti* ES proteins with *C. elegans*, parasitic nematodes and rat (host) protein sequence databases using SimiTri.** The numbers at each vertex indicate the number of proteins matching only that specific database. The numbers on the edges indicate the number of proteins matching the two databases linked by that edge. The number within the triangle indicates the number of S. ratti ES proteins with matches to all three databases.

2310 (89.8%) *S. ratti* ES proteins had homologues in the free-living nematode, *C. elegans*. 2220 (86.3%) ES proteins had homologues in parasitic nematodes. As *S. ratti* infects rats, we checked the similarity of ES proteins with the rat proteome. Similarity of *S. ratti* ES proteins to *C. elegans*, parasitic nematodes and rat proteins is shown using Simitri in Figure 2. We found 537 (20.8%) ES proteins had no homologues present in rat and are therefore preferred targets for parasite intervention strategies. 142 ES proteins are novel in the *S. ratti* dataset, with no known homologues to the host or any other nematode. 233 (9%) ES proteins, which are not present in the host (rat), have homologues present in *C. elegans*. Of these, 19 ES proteins (predicted from second order contigs from CAP3 assembly), which have lethal RNAi phenotypes present in *C. elegans*, (complete RNAi phenotype mapping available from Additional file 4) and represent potential therapeutic targets (Additional file 5).

## Discussion

We demonstrated the utility of our new computational approach for the comprehensive prediction and analysis of ES proteins from transcriptomic data generated by NGS. The protocol will be implemented in a web server, in the future, after extensive testing of different assembly programs, and considering the choice of specific assemblers, based on the transcriptomic dataset, as proposed by Kumar and Blaxter [10]. For this study, we have selected programs that are freely available under academic licence. All the programs used in our approach are available with free academic licence, which can be easily installed on Linux platforms. Our use of MIRA followed by CAP3 for assembly of NGS data is simpler than the assembler combinations proposed by Kumar and Blaxter [10] and also used by studies on *Fasciola hepatica *[*12*], *Clonorchis sinensis *[*18*] and *Opisthorchis viverrini*[*18*] to generate second order contigs by CAP3 from contigs generated by MIRA which have open reading frames. The whole assembly for the current dataset was performed in approximately 3 hours CPU time using both MIRA and CAP3, whereas the use of CAP3 alone was not possible due to memory overflow with the current dataset, using hardware specified in the methods section. Although all the studies discussed here are more comprehensive in terms of transcriptome coverage (more than 0.5M 454 reads were generated), which is higher as compared to our current dataset of ~0.3M, none of them have comprehensively studied ES proteins. For example, the 454 transcriptomic study on *Fasciola hepatica *[*12*] reported only 1812 ES proteins (only 4%) from 44597 putative protein sequences generated from ESTScan, followed by ES protein predictions based on signal peptide identification by SignalP.

### Biological implications of the results

Millions of people globally suffer from Strongyloidiasis, caused by the parasitic nematode, *Strongyloides stercoralis. S. ratti* is a common gastro-intestinal parasite of the rat, which is used as a model to study Strongyloidiasis. Here, we have analysed *S. ratti* transcriptomic data from parasitic females, free-living males and free-living females for the prediction and analysis of ES proteins. Of the dataset of 2572 ES proteins 2310 (89.8%) had homologues in the free-living nematode, *C. elegans*, which is similar to earlier reported findings in Strongyloides EST analysis studies [59]*.* Many predicted ES proteins map to protein kinase domains as shown in Table 2, which are reported to be essential for parasitic activity in parasitic nematodes [60]. Protein kinases play a central role in signal transduction and hence are considered as drugabble targets. Another representative Interpro protein domains among *S. ratti* ES proteins were WD40 repeat domains (7.5%), which are associated with signalling transduction pathways [61]. These domains were also found among the top 20 most represented Interpro protein domains of *O. dentatum* putative proteins [14]. ES proteins also map to ribosomal protein interpro domains such as IPR000589 (Ribosomal protein S15), which is associated with ageing in *S. ratti *[62]. All the most representative KEGG pathways mapped to ES proteins shown in table 3 are required for parasite survival inside the host, as the secretome of a parasite is representative of its genome in the host environment. Major ES proteins map to enzymes, which are essential for metabolic pathways functioning and also very well reflected in our protein domain mapping. Other KEGG pathways like purine metabolism and glutathione metabolism found in this study were also found in other parasitic nematodes excretory/secretory proteins analysis [23]. 22 (0.85%) ES proteins were mapped to the circadian rhythm – mammal pathway in *C. elegans*. This pathway is unexpected in the case of ES proteins of nematodes, however three proteins S-phase kinase-associated protein 1 (KO3094), cullin 1 (KO3347) and F-box and WD-40 domain protein 1/11 (KO3362) which were found in our ES proteins are common to Ubiquitin mediated proteolysis in *C. elegans*. The common components of several pathways have led to this unexpected result. KEGG BRITE objects (representative objects shown in Table 4) reflect the presence of essential proteins such as protein kinases, peptidases and proteasome among ES proteins for *S. ratti* survival inside the host organism*.* 44 (1.71%) ES proteins map to chaperones, which are responsible for host immune system modulation, such as the recently characterised *S. ratti* heat shock protein 10 [63]. Along with well known protein families found in ES proteins, we found some protein categories such as chromosome, DNA replication proteins and DNA repair and recombination proteins which are expected to be localized in the nucleus but found in *S. ratti* ES proteins. This pattern of exporting nuclear proteins to the secretome of a parasitic nematode was also observed in *Meloidogyne incognita *[64]. 66 secreted proteins were identified with putative nuclear localization such as DNA and RNA binding proteins including helicases in *M. incognita*, of which we observed the presence of helicase C domain in 35 (1.36%) *S. ratti* ES proteins. Contig 1289 and Contig 428 map to the metalloproteinase precursor in *S. stercoralis *[*65*], this is also well characterized protein in *Trichinella spirallis*[66]. Expresssion of an *S. stercoralis* metalloproteinase homologue was also found in the recent transcript analysis of another intestinal nematode, *Strongyloides venezuelensis *[67]. Many of these potential therapeutic targets map to hypothetical proteins present in *C. elegans*, *C. briggsae* and *B. malayi* and having lethal phenotypes according to *C. elegans* RNAi phenotype mapping and could be considered as parasitism central genes [68] of *S. ratti*. Many of the putative proteins from *S. ratti* could be examined further after the publication of *S. ratti* genome, which is expected soon [69].

### Methodological limitations

Integrated approaches similar to the one discussed in this paper have been applied to several socio-economically important parasites. These approaches are based on data available on the reference organism of that taxonomic order where limited data is available for the subject organism. For example, *C. elegans* is the most studied organism among nematodes. *C. elegans* data was used to create the translation matrix used by ESTScan, to translate potential coding regions in the assembled contigs into protein sequences. These translated coding regions were then used for ES proteins prediction. The use of a reference organism data for the translation matrix instead of using actual organism information may lead to false positives in peptides prediction as well as in ES protein prediction. Another limiting factor is that we are looking into the annotation of protein function in terms of primary sequence alone, rather than the 3D structure. Therefore, all the therapeutic targets predicted in this study are preliminary predictions which need to be further validated by additional computation analysis such as structural modelling and by experimental assays.

## Conclusions

In this paper we demonstrate how different computational tools can be used together to extract the useful information of ES proteins from transcriptomic data. All the programs used in our approach are open source tools that are freely available for academic purposes. With the advent of NGS technologies, while there is a massive increase in sequence data, this data is extremely fragmented and of no use for information extraction as output from the sequencer. Our methodology will help in rapid assembly, fast annotation and reliable prediction of ES proteins. The approach is a generalized method which can be applied to any organism, although its main application is for neglected organisms whose genomes are not yet sequenced, with limited functional knowledge. Although we have used 454 transcriptomic data in this study but this methodology can be applied to transcriptomic data from other NGS platforms with slight modifications in terms of pre-processing, as data output formats obtained from different NGS platforms are different. Thus, this system will help us to carry out secretome studies for other parasitic organisms in future.

## List of abbreviations used

BRITE: Biomolecular Relations in Information Transmission and Expression; KEGG: Kyoto Encyclopedia of Genes and Genomes; KAAS: KEGG automatic annotation server.

## Competing interests

The authors declare that they have no competing interests.

## Authors’ contributions

SR directed the study. GG did the analysis. SR and GG contributed to writing the manuscript.

## Supplementary Material

Additional file 1 **Protein domain mapping of *S. ratti* ES proteins**. Represented Interpro domains found in *S. ratti* ES proteins using Interproscan (sheet1). Protein domains mapping of *S. ratti* excretory/secretory proteins (sheet2). Click here for file

Additional file 2 **KEGG pathways mapping of *S. ratti* ES proteins.** Represented KEGG pathways found in ES proteins predicted by KAAS (Table S2). Click here for file

Additional file 3 **KEGG BRITE objects mapping of *S. ratti* ES proteins.** Represented KEGG BRITE objects found in ES proteins predicted by KAAS (Table S3). Click here for file

Additional file 4 **RNAi Phenotype mapping of *S. ratti* ES proteins**. RNAi Phenotype mapping of *S. ratti* ES proteins against known *C. elegans* known phenotypes (sheet1). Click here for file

Additional file 5 **Representative therapeutic targets set of *S. ratti* ES proteins.** Representative therapeutic targets set of *S. ratti* ES proteins, homologous to *C. elegans* proteins with lethal RNAi phenotype and with no homologue in the host, rat*.* *Click here for file*
