# Supplementary material for: In silico secretome analysis approach for next generation sequencing transcriptomic data
Source: BMC Genomics. 2011 Nov 30;12(Suppl 3):S14. doi: 10.1186/1471-2164-12-S3-S14 (PMC3333173; doi:10.1186/1471-2164-12-S3-S14)
Supplement: Additional file 2 — KEGG pathways mapping of S. ratti ES proteins. Represented KEGG pathways found in ES proteins predicted by KAAS (Table S2). [file 1471-2164-12-S3-S14-S2.pdf]

Additional File 2: ***In silico*** **secretome analysis approach for next generation sequencing transcriptomic data**

Gagan Garg and Shoba Ranganathan

Table S2 -Represented KEGG pathways found in ES proteins predicted by KAAS

Pathway name	Number of ES proteins represented
Metabolic pathways	109
Protein processing in endoplasmic reticulum	57
Ubiquitin mediated proteolysis	44
Wnt signalling pathway	29
Glycolysis / Gluconeogenesis	28
Spliceosome	28
Glutathione metabolism	26
Circadian rhythm - mammal	22
TGF- beta signalling pathway	22
RNA transport	20
Endocytosis	20
Purine metabolism	19
Phagosome	19
Proteasome	18
Drug metabolism	17
MAPK signaling pathway	17
Lysosome	16
Arginine and proline metabolism	16
Pyrimidine metabolism	15
Pyruvate metabolism	15
Amino sugar and nucleotide sugar metabolism	14
Aminoacyl-tRNA biosynthesis	13
Metabolism of xenobiotics by cytochrome P450	13
Valine, leucine and isoleucine degradation	13
Alanine, asparatate and glutamate metabolism	12
Calcium signaling pathway	12
Propanoate metabolism	12
Phosphatidylinositol signaling system	11
mRNA surveillance pathway	11
Pentose phosphate pathway	10
Fructose and mannose metabolism	9
RNA degradation	9
Arachidonic acid metabolism	9
Beta-Alanine metabolism	9
TCA cycle	9
Glycerophospholipid metabolism	9
Glycerolipid metabolism	8
Starch and sucrose metabolism	8
Fatty acid metabolism	8

Oxidative phosphorylation	7
Hedgehog signaling pathway	6
Progesterone-mediated oocyte maturation	6
ErbB signaling pathway	6
Nucleotide excision repair	6
Nitrogen metabolism	6
Galactose metabolism	6
Tryptophan metabolism	5
Histidine metabolism	5
DNA replication	5
Valine, leucine and isoleucine biosynthesis	5
Inositol phosphate metabolism	5
Basal transcription factors	5
Lysine degradation	5
Ribosome	5
mTOR signaling pathway	4
Taurine and hypotaurine metabolism	4
Ascorbate and aldarate metabolism	4
N-Glycan biosynthesis	3
Cyanoamino acid metabolism	3
Jak-STAT signaling pathway	3
Glyoxylate and dicarboxylate metabolism	3
Regulation of autophagy	3
Riboflavin metabolism	3
Ribosome biogenesis in eukaryotes	3
Notch signaling pathway	3
Ether lipid metabolism	3
Pentose and glucuronate interconversions	3
Butanoate metabolism	3
Base excision repair	3
Peroxisome	3
Other types of O-glycan biosynthesis	2
Sphingolipid metabolism	2
Fatty acid biosynthesis	2
Selenocompound metabolism	2
Drug metabolism – other enzymes	2
Porphyrin and chlorophyll metabolism	2
Cysteine and methionine metabolism	2
Sulfur relay system	2
Mismatch repair	2
Glycine, serine and threonine metabolism	2
Other glycan degradation	2
RNA polymerase	1
Glycosaminoglycan degradation	1
Nicotinate and nicotinamide metabolism	1
Lysine biosynthesis	1
ECM-receptor interaction	1
Glycosylphosphatidylinositol (GPI)-anchor biosynthesis	1

Lipoic acid metabolism	1
Natural killer cell mediated cytotoxicity	1
Dorso-ventral axis formation	1
Phenylalanine metabolism	1
Tyrosine metabolism	1
Phenylalanine, tyrosine and tryptophan biosynthesis	1
D-Glutamine and D-glutamate metabolism	1
Glycosphingolipid biosynthesis- ganglio series	1
