# Supplementary material for: In silico secretome analysis approach for next generation sequencing transcriptomic data
Source: BMC Genomics. 2011 Nov 30;12(Suppl 3):S14. doi: 10.1186/1471-2164-12-S3-S14 (PMC3333173; doi:10.1186/1471-2164-12-S3-S14)
Supplement: Additional file 3 — KEGG BRITE objects mapping of S. ratti ES proteins. Represented KEGG BRITE objects found in ES proteins predicted by KAAS (Table S3). [file 1471-2164-12-S3-S14-S3.pdf]

Additional File 3: In silico secretome analysis approach for next generation sequencing transcriptomic data

Gagan Garg and Shoba Ranganathan

Table S3- Represented KEGG BRITE objects found in ES proteins predicted by KAAS

BRITE object	Number of ES proteins represented
Enzymes	282
Spliceosome	49
Chaperons and folding catalysts	44
Peptidases	44
Protein kinases	43
Ubiquitin system	37
Chromosome	34
Cytoskeleton proteins	27
DNA repair and recombination proteins	21
GTP-binding proteins	19
Proteasome	18
Transcription factors	17
Ribosome biogenesis	16
Translation factors	11
DNA replication proteins	9
Transporters	8
Glycan Binding proteins	7
Cellular antigens	7
Glycosyltransferases	6
Secretion system proteins	5
CAM ligands	5
Ion Channels	4
Ribosome	4
Bacterial motility proteins	3
Lipid biosynthesis proteins	2
Proteoglycans	2
Cytokines	2
Nuclear receptors	1
G protein-Coupled Receptors	1
Cell adhesion molecules (CAMs)	1
