# Supplementary material for: In silico secretome analysis approach for next generation sequencing transcriptomic data
Source: BMC Genomics. 2011 Nov 30;12(Suppl 3):S14. doi: 10.1186/1471-2164-12-S3-S14 (PMC3333173; doi:10.1186/1471-2164-12-S3-S14)
Supplement: Additional file 5 — Representative therapeutic targets set of S. ratti ES proteins. Representative therapeutic targets set of S. ratti ES proteins, homologous to C. elegans proteins with lethal RNAi phenotype and with no homologue in the host, rat. [file 1471-2164-12-S3-S14-S5.pdf]

Additional File 5: In silico secretome analysis approach for next generation sequencing transcriptomic data

Gagan Garg and Shoba Ranganathan

Table S5-Representative therapeutic targets set of *S. ratti* secretory proteins, homologous to *C. elegans* proteins with lethal RNAi phenotype and with no homologue in the host, rat.

No.	Cluster ID	E value	Identity (%)	Description from NR database	WBGene ID	*C. elegans* homologue	*C. elegans* RNAi phenotype	*C. elegans* gene ontology
1	Contig1034;	1.00E-09	32.7	UNC-34 [*Caenorhabditis elegans*]	WBGene00006770	CE43197	cell migration variant, locomotion variant	CELLULAR COMPONENT<br>filopodium tip cell junction<br>actin cytoskeleton<br>BIOLOGICAL PROCESS<br>cell adhesion<br>cell migration<br>MOLECULAR FUNCTION<br>hormone activity<br>structural constituent of cell wall
2	Contig1295;	1.00E-21	56.63	FK506-Binding protein family member (fkb-3) [*Caenorhabditis elegans*]	WBGene00001428	CE07921	dauer development variant	CELLULAR COMPONENT<br>endoplasmic reticulum<br>BIOLOGICAL PROCESS<br>protein folding determination of adult life span
3	Contig1718;	2.00E-140	53.33	Lin-5 (Five) interacting protein family member (lfi-1) [*Caenorhabditis elegans*]	WBGene00022500	CE31264	embryonic lethal	CELLULAR COMPONENT<br>kinetochore microtubule<br>MOLECULAR FUNCTION<br>transcription factor activity<br>sequence-specific DNA binding<br>protein dimerization activity<br>signal transducer activity<br>BIOLOGICAL PROCESS<br>regulation of transcription, DNA-dependent<br>signal transduction
4	Contig1740;	4.00E-15	60.92	Hypothetical protein [*Brugia malayi*]	WBGene00001561	CE34603	organism hypertonic lethality increased, ectopic expression	BIOLOGICAL PROCESS<br>filament organization

							transgene, multivulva, sterile, sick, larval lethal, maternal sterile, protruding vulva , pattern of transgene expression variant, receptor mediated endocytosis defective, lethal, embryonic lethal, slow growth, transgene expression increased	hermaphrodite genitalia development<br>regulation of vulval development<br>CELLULAR COMPONENT<br>intermediate filament<br>MOLECULAR FUNCTION<br>structural constituent of cytoskeleton protein binding
5	Contig1795;	7.00E-21	49.12	Hypothetical protein C01F1.1 [*Caenorhabditis elegans*]	WBGene00015296	CE06742	sterile, pattern of transgene expression variant, receptor mediated endocytosis defective protruding vulva, slow growth, age associated fluorescence increased ,embryonic lethal, reduced brood size, shortened life span, sterile, protruding vulva	MOLECULAR FUNCTION<br>RNA polymerase II transcription factor activity<br>CELLULAR COMPONENT<br>nucleus<br>BIOLOGICAL PROCESS<br>positive regulation of transcription<br>embryonic development<br>transcription initiation from RNA polymerase II promoter
6	Contig1865;	1.00E-32	61.32	Major sperm protein [*Brugia malayi*]	WBGene00006047	CE09025	transgene expression reduced	MOLECULAR FUNCTION<br>structural molecule activity
7	Contig2131;	8.00E-17	53.57	Myosin heavy chain, nonmuscle type 1 [*Brugia malayi*]	WBGene00003776	CE31177	embryonic lethal, organism morphology variant, dumpy, locomotion variant, sterile progeny,, larval arrest	BIOLOGICAL PROCESS<br>shape changes of embryonic cells nematode larval development<br>protein localization<br>signal transduction<br>body morphogenesis<br>collagen and cuticulin-based cuticle development<br>CELLULAR COMPONENT<br>contractile fiber<br>cell-cell adherens junction<br>myosin complex<br>MOLECULAR FUNCTION<br>motor activity

								ATP binding
8	Contig2153;	3.00E-50	44.2	C. briggsae CBR-TRY-10 protein [*Caenorhabditis briggsae*]	WBGene00045418	CE41117	fat content increased	CELLULAR COMPONENT integral to membrane
9	Contig2161;	8.00E-17	71.19	Triosephosphate isomerase [*Caenorhabditis. elegans*]	WBGene00006601	CE19040	body wall muscle myosin organization defective, shortened life span	MOLECULAR FUNCTION triose-phosphate isomerase activity BIOLOGICAL PROCESS metabolic process
10	Contig2465;	2.00E-17	48.42	Hypothetical protein F53B6.4 [*Caenorhabditis elegans*]	WBGene00009959	CE10896	locomotion variant	MOLECULAR FUNCTION structural molecule activity CELLULAR COMPONENT integral to membrane
11	Contig2583;	9.00E-16	38.83	Hypothetical protein CBG03803 [*Caenorhabditis briggsae*]	WBGene00009160	CE32638	cord commissures fail to reach target, ventral cord patterning variant, axon midline crossing variant	BIOLOGICAL PROCESS protein amino acid phosphorylation MOLECULAR FUNCTION protein kinase activity ATP binding protein binding
12	Contig2671;	1.00E-23	53.27	*Sperm-specific family, class P family member (ssp-10)*[*Caenorhabditis elegans*]	WBGene00006040	CE09149	transgene expression reduced, fat content increased	MOLECULAR FUNCTION structural molecule activity
13	Contig2766;	2.00E-52	53.42	RE06140p [*brugia malayi*]	WBGene00011883	CE03683	larval arrest, slow growth, embryonic lethal, small, clear, embryonic lethal	BIOLOGICAL PROCESS positive regulation of growth rate embryonic development ending in birth or egg hatching nematode larval development
14	Contig29;	2.00E-09	29.55	Hypothetical protein CBG18079 [*Caenorhabditis briggsae*]	WBGene00015049	CE06688	fat content reduced	MOLECULAR FUNCTION protein kinase activity ATP binding BIOLOGICAL PROCESS protein amino acid phosphorylation
15	Contig2903;	6.00E-15	27.12	TsJ5 [*Trichinella spiralis*]	WBGene00019510	CE18030	transgene induced cosuppression variant, slow growth, embryonic	MOLECULAR FUNCTION nucleotide binding

							lethal	BIOLOGICAL PROCESS positive regulation of growth rate embryonic development ending in birth or egg hatching
16	Contig2930;	3.00E-33	48.82	Hypothetical protein Bm1_55970 [*Brugia malayi*]	WBGene00016015	CE25784	transgene expression increased, maternal sterile, protruding vulva,slow growth, embryonic lethal, maternal sterile	BIOLOGICAL PROCESS embryonic development ending in birth or egg hatching positive regulation of growth rate hermaphrodite genitalia development
17	Contig506;	8.00E-13	37.29	Hypothetical protein F53B6.4 [*Caenorhabditis elegans*]	WBGene00009959	CE10896	locomotion variant	MOLECULAR FUNCTION structural molecule activity CELLULAR COMPONENT integral to membrane
18	Contig858;	2.00E-13	46.15	Predicted: upstream activation factor subunit spp27 – like [*Apis mellifera*]	WBGene00020779	CE37993	embryonic lethal	CELLULAR COMPONENT Nucleus BIOLOGICAL PROCESS embryonic development ending in birth or egg hatching
19	Contig914;	6.00E-127	64.65	Hypothetical protein T19B10.2 [*Caenorhabditis elegans*]	WBGene00011831	CE16413	reduced brood size, age associated fluorescence increased, larval lethal, larval arrest, locomotion variant, paralyzed organism osmotic stress response variant, molt defect , shortened life span, slow growth, exploded through vulva, locomotion variant, transgene subcellular localization variant, protein aggregation variant	BIOLOGICAL PROCESS locomotion positive regulation of growth rate morphogenesis of an epithelium molting cycle, collagen and cuticulin-based cuticle
